# Intranasal Vaccine Delivery Technology for Respiratory Tract Disease Application with a Special Emphasis on Pneumococcal Disease

**DOI:** 10.3390/vaccines9060589

**Published:** 2021-06-02

**Authors:** William Walkowski, Justin Bassett, Manmeet Bhalla, Blaine A. Pfeifer, Elsa N. Bou Ghanem

**Affiliations:** 1 Department of Chemical and Biological Engineering, University at Buffalo, The State University of New York, Buffalo, NY 14260, USA; wewalkow@buffalo.edu (W.W.); jdbasset@buffalo.edu (J.B.); blainepf@buffalo.edu (B.A.P.); 2 Department of Microbiology and Immunology, University at Buffalo, The State University of New York, Buffalo, NY 14203, USA; manmeetp@buffalo.edu

**Keywords:** intranasal, pulmonary, respiratory tract, vaccine, pneumococcal disease, aging

## Abstract

This mini-review will cover recent trends in intranasal (IN) vaccine delivery as it relates to applications for respiratory tract diseases. The logic and rationale for IN vaccine delivery will be compared to methods and applications accompanying this particular administration route. In addition, we will focus extended discussion on the potential role of IN vaccination in the context of respiratory tract diseases, with a special emphasis on pneumococcal disease. Here, elements of this disease, including its prevalence and impact upon the elderly population, will be viewed from the standpoint of improving health outcomes through vaccine design and delivery technology and how IN administration can play a role in such efforts.

## 1. Introduction

Respiratory tract infectious diseases are ubiquitous due to the airways being the most accessible route to bodily entry. Subsequent infections of the ears, nose, throat, and lungs produce a range of symptoms associated with both viral and bacterial pathogens [1,2,3,4]. Such illnesses are also readily transmissible via aerosolization [5].

Primary examples of respiratory tract diseases include the yearly occurrences of influenza, pneumonia, and the common cold [5,6]. In particular, secondary bacterial pneumonia (commonly triggered by influenza co-infection) can have devastating impacts on the very young and elderly populations, with the resulting illness highlighting synergy between bacterial and viral pathogens [6,7,8,9,10]. Of course, the events of 2020 highlight the continued emergence of coronaviruses into the public perception of respiratory tract diseases.

Various therapies over the years have been applied and tested for respiratory tract diseases, with an emergence of prophylactic options for both viral and bacterial infectious agents [11,12,13,14,15]. A key element of this mini-review will be to present and analyze the preventative options, via vaccination, available to common respiratory tract diseases and how such treatment options are designed and delivered.

In particular, we will closely examine the option of intranasal (IN) delivery for respiratory tract illnesses. IN administration offers numerous advantages to the delivery of both therapeutics and prophylactics due to the obvious co-localization of the treatment proximal to infection.

Based upon current collaborative efforts of the authors, we will also more closely examine pneumococcal disease as an important respiratory tract illness, especially as it relates to the elderly population, defined as individuals > 65 years old, a group that will greatly expand in size over the next 25 years [16]. The unique features of pneumococcal disease, its relationships to other respiratory tract illnesses (particularly influenza), and the consideration that must be given to vaccine development will be highlighted. Included in this analysis will be the potential role which IN vaccine delivery might serve.

As such, we will intertwine the impact of respiratory tract diseases, some of the key elements of disease progression, and the application of IN delivery in the treatment of these diseases. We will also link the IN delivery approach to elements of particular respiratory tract illnesses (focusing on pneumococcal disease) and how this administration route can facilitate immune reactivity, or effective treatment more generally, towards the foundations of disease and disease progression.

## 2. Main Respiratory Illnesses and Their Treatment Methods

Respiratory tract infectious diseases have a long history of affecting the quality of human life. In this section, we introduce this disease category more broadly, highlighting high profile examples, their historical and recent impact, and treatment options available (Table 1).

In Table 1, well-recognized infectious diseases are highlighted, all of which have direct impact upon the respiratory tract. Some of these diseases are relatively common and mild (i.e., influenza and the common cold); however, certain viral strains over time have had significant global impact, especially as it relates to flu pandemics. Other diseases, like pneumococcal disease and tuberculosis, are caused by bacterial pathogens. There is also the potential interplay between various respiratory tract diseases, such as that between pneumococcal disease and influenza, which will be discussed in greater detail later.

Table 1 also presents common treatment options for the pulmonary diseases listed. Notably, each disease includes a vaccine option, even those that are bacterial in nature and might be readily treated by antibiotics. In the case of bacterial-derived tuberculosis, antibiotic effectiveness can be limited by the latent state of the bacteria and/or the morphology and chemical composition of the mycobacterium responsible for disease [23,24,25,26,27]. The disease progression profile for pneumococcal disease (namely, in vivo biofilm formation) also poses challenges for effective antibiotic treatment [28,29,30,31,32,33]. Finally, like most other bacterial infectious disease targets, the active agents responsible for tuberculosis, pertussis, and pneumococcal disease are prone to development of antibiotic resistance [34,35,36,37,38,39]. As a result, all of the respiratory tract diseases listed in Table 1 also feature vaccine treatment options, with each of these routinely used to address the given disease.

## 3. Intranasal Vaccine Delivery

Table 1 includes vaccines as a consistent treatment option for the range of respiratory tract diseases highlighted. In this section, we will now introduce intranasal delivery as a vaccine administration route. As the name indicates, intranasal delivery means a vaccine formulation, which is introduced in the body through the nose. In the context of treatment options for respiratory tract diseases, this approach is viewed with great promise.

First, when compared to more traditional forms of vaccine administration methods (subcutaneous or intramuscular injection), intranasal administration offers a simpler, less invasive option, potentially leading to more compliance and less medical complications (localized infection and/or pain) due to traditional methods that require needle-based skin puncture [40], though it is acknowledged that overall effectiveness will depend on the consistent degree of IN administration, which is subject to the methodology of delivery or the skill of personnel overseeing the administration. In addition, in the context of vaccine delivery for the aforementioned respiratory tract diseases, intranasal delivery offers the prospect of a localized, mucosal immune response proximal to the target infectious disease [40,41].

Table 2 covers the broad use of intranasal delivery methods applied to the infectious diseases introduced in Table 1 (with the majority of these studies reporting similar or better results compared to traditional methods of intramuscular and subcutaneous administration). Spacing the analysis over a 12-year window, the total number of IN vaccine studies completed over this time period were directed at influenza. Whooping cough (pertussis) has a very established vaccine treatment regimen, and this may decrease newer research efforts for IN delivery methods. Whereas, diseases like influenza, pneumococcal disease, and tuberculosis feature some combination of challenging disease features, whether that be seasonal strain variation (influenza), degree of strain coverage and disease progression (pneumococcal disease), or disease state (active vs. latent, in the case of tuberculosis). As such, the complexities associated with these disease types may have spurred more recent research activity more generally with the inclusion of IN vaccine delivery methods in particular. Finally, the dominance in attention due to COVID-19 research over the last year offers an explanation for the recent uptick in IN delivery research [42] (with a noticeable downtrend for studies focused on influenza in that same time period).

Table 3 more closely examines the types of IN methods that have been utilized over the last 10 years, the rationale behind the methodology, general frequency of use, and distribution of application across common respiratory tract diseases. The approaches highlighted include direct IN administration (the addition/inhalation of a dry powder or instillation within a solution applied directly to the nostrils) and the inclusion of both physical (aerosolization) and chemical (various formulations) methods designed to influence bodily absorption and immune response. Applications generally span the respiratory tract diseases introduced in Table 1 and Table 2 with a heightened degree of usage for influenza and pneumococcal disease.

## 4. Pneumococcal Disease and the Elderly

The following section will feature an extended examination of pneumococcal disease, due both to overlapping collaborative expertise on the part of the authors and its connection to the themes of this mini-review. Of note, pneumococcal disease, derived from *Streptococcus pneumoniae*, is particularly relevant due to its broad global impact, especially on the very young, elderly, and resource limited [54,55,56,57]; its unique means of disease progression spanning the upper respiratory tract to various downstream locations in the body (including and prominently the lungs) [33,58]; its potentially devastating overlap with dual infectious diseases, predominantly influenza [9,10,55,59,60,61]; and the opportunity to address this disease through unique means of vaccine design and delivery, including intranasal administration.

Pneumococcal disease has a disproportional impact upon the elderly, where individuals ≥ 65 years old account for the majority of hospitalizations and deaths following pneumococcal infection [62]. The number of elderly is projected to double in the coming decades, reaching 2 billion by 2050 [63]. This poses a serious health concern as the elderly are more susceptible to infections, particularly those caused by *S. pneumoniae* [54], which are encapsulated Gram-positive bacteria that include 100 serotypes based on the composition of the capsular polysaccharide [62,64]. Upon colonization of the upper respiratory tract, these bacteria typically reside asymptomatically in the nasopharynx of healthy individuals [62], occurring in 10–40% of adults and up to 80–100% in children [65]. In individuals with compromised immunity, such as the elderly, pneumococci can spread and cause pneumonia as well as invasive pneumococcal diseases, including meningitis, endocarditis, and bacteremia [54]. Disease manifestation is in part driven by bacterial serotypes, as bacteria with different capsular polysaccharides vary considerably in their ability to cause invasive disease [66]. Despite available vaccine and antibiotic treatments, *S. pneumoniae* remain the leading cause of bacterial community-acquired pneumonia in the elderly [67] and according to the CDC are responsible for 900,000 cases of pneumonia and 400,000 hospitalizations in the U.S. yearly [68]. In a recent Active Bacterial Core surveillance report [69], individuals above 50 accounted for 71% of all invasive pneumococcal diseases cases and 82% of associated deaths [70,71], resulting in an estimated cost of $2.5 billion annually due to hospitalizations [72,73]. Of further concern is the increase in pneumococcal antibiotic resistance (thus, limiting traditional antibiotic use), classified by the 2019 CDC Antibiotic Resistance Threat Report as *Serious* and resulting in over a million drug-resistant infections yearly [74]. Strikingly, the elderly are more at risk of acquiring drug-resistant infections [68]. The risk of pneumococcal pneumonia is further enhanced dramatically (100-fold) by influenza A virus (IAV) co-infection [60,75], resulting in seasonal increases in lethal infections, the majority of which (70–85%) are in elderly individuals [60]. Without intervention, projected increases in the aging population will double pneumococcal-related health impacts and treatment costs in the coming decades [72], necessitating novel strategies to combat this infectious threat.

Two vaccines consisting of capsular polysaccharides that cover the most common disease-causing *S. pneumoniae* serotypes are recommended for the elderly [76]. The pneumococcal polysaccharide vaccine (PPSV or Pneumovax) covers 23 serotypes and triggers T cell-independent antibody (Ab) production with 56–75% efficacy (in non-elderly groups). The pneumococcal conjugate vaccine (PCV or Prevnar-13) contains polysaccharides from 13 strains covalently linked to a non-pathogenic diphtheria toxoid protein (CRM197) that triggers a T cell-dependent antibody response [76]. PCV provides protection against 74–88% of invasive pneumococcal disease cases (in non-elderly groups). The introduction of PCV in children leads to eradication of bacterial nasal colonization or carriage, thereby, reducing transmission and indirectly leading to a decline in infections within adults for strains included in the vaccine.

However, there are several issues associated with the currently licensed vaccines that limit their efficacy (defined as prevention of infection by pneumococci) against pneumococcal infections overall and particularly in the elderly population. The first is serotype coverage and replacement [77,78]. The above vaccine methods focused upon inhibiting initial colonization have prompted increased infections caused by “replacement” strains not included in the current vaccines. This prospect has been made more daunting by the sizable number of serotypes (currently 100 identified thus far [64]) that must be accounted for to enable full vaccine coverage [79,80]. Moreover, novel disease-associated non-encapsulated pneumococcal strains that carry antibiotic resistance genes have recently emerged [81], and these are not covered by the available licensed vaccines. The second issue with current vaccines is a failure to account for changes in pneumococcal biology during disease progression. *S. pneumoniae* typically reside asymptomatically in the nasopharynx of healthy individuals [62], and it is hypothesized that *S. pneumoniae* establish an asymptomatic biofilm on the nasopharyngeal epithelium by attenuating the production of virulence factors and concomitant inflammation [32,82,83,84]. In humans, pneumococcal carriage is believed to be a prerequisite of invasive disease [85,86], which occurs when immunity is compromised, as is observed in the elderly. The transition from benign colonizer to lethal pulmonary or systemic pathogen also involves changes in bacterial transcript profiles and morphology [83,87,88,89]. This was highlighted in recent studies that showed that the set of genes expressed by pneumococci during colonization were distinct from those expressed during lung infection as well as during bacteremia, indicating that the bacteria adapt to their host in an infection site/organ-specific manner [83,87]. Importantly, sets of conserved genes were upregulated across the several strains tested, suggesting they could be potential vaccine targets that induce strain-independent protection [87,89]. Similarly, it is well-established that regulation of capsule expression is required for bacterial virulence [90]. Capsule expression is required for evasion of entrapment by mucus in the airways; however, downregulation of capsule allows for efficient bacterial binding to the pulmonary epithelium [91]. Upon bacterial localization into deeper tissues, including the lower airways and the bloodstream, capsule formation is again required to evade phagocytosis and clearance by immune cells [80,91]. These findings have important implication on vaccine design, and vaccines that encompass capsular polysaccharides along with other bacterial factors key for establishment of lung infection or invasive disease (e.g., bacteremia) would be ideal for eliciting full host protection against infection. Finally, the third issue with current vaccines is reduced efficacy during aging. PPSV has been traditionally recommended for the elderly while PCV is now recommended for the most vulnerable elderly with underlying conditions [79,92]. While protective against bacteremia, the efficacy of both vaccines is limited against pneumonia in the elderly: PPSV and Prevnar-13 showed only 33% [93] and 45% protection against pneumonia, respectively [94,95]. This age-driven decline in pneumococcal immunization and conjugate vaccine efficacy has been recapitulated in mice [96,97]. The moderate ability of current vaccines in protecting against pneumonia in the elderly necessitates better strategies to boost vaccine efficacy.

Immunosenescence, the overall dysregulation in immunity that occurs with age, drives the increased susceptibility of the elderly to invasive pneumococcal diseases and the linked decline in vaccine efficacy [98,99,100]. Several aspects of the age-related decline in adaptive immunity have been characterized [97,101,102]. Antibody production by B cells can depend on T cells such as that elicited by PCV [103] or be T cell-independent as elicited by PPSV [104]. Aging leads to defects in both T cell-dependent and -independent antibody production [105,106], limiting current vaccine efficacy [105,107]. Following vaccination with PPSV, both antibody levels and functionality, defined as the ability of antibodies to opsonize and enhance phagocytic uptake of bacteria (opsonophagocytic activity or OPA), were significantly impaired among the elderly when compared to younger individuals [108]. The drivers of declined vaccine response in aging are multi-factorial and may be attributed to chronic inflammation [105], intrinsic defects in B cells including reduced repertoire, defects in key transcription factors and reduction in AID, the enzyme required for class-switch recombination and somatic hypermutation, as well as overall defects in T cell signaling and proliferation [76,109] and in T-follicular helper cells that mediate antibody production by B cells [110,111]. Thus, vaccines that enhance antigen presentation and simultaneously target more than one arm of the immune response are attractive avenues to boost memory responses in the elderly.

In the U.S., influenza accounts for over 10,000 deaths annually and over 40,000 deaths during epidemic years [112]. Individuals ≥ 65 years account for a staggering 88% of all influenza-associated deaths [112]. A high percentage of deaths during major influenza pandemics are due to secondary bacterial pneumonia, particularly by *S. pneumoniae* [113]. In fact, the risk of invasive pneumococcal infection is enhanced 100-fold by influenza A virus (IAV) infection [112], resulting in the seasonal peak of invasive pneumococcal disease during influenza outbreaks [60]. The means by which IAV promotes bacterial infection are manifold and have been characterized using mouse models of co-infection [114]. As mentioned, *S. pneumoniae* typically colonizes asymptomatically, and it is thought that IAV infection triggers bacterial release from the nasopharynx into the lungs, priming the infection [115]. First, IAV exposure enhances the nutritional environment for pneumococcus in the nasopharynx by increasing the availability of sialylated substrates and increasing rates of pneumococcal carriage [116]. Second, factors such as ATP, released by viral infected host cells, promote the dispersion of pneumococci from nasopharynx biofilms to the lower respiratory tract [83,116,117]. The dispersed bacteria have altered transcriptional profiles and express increased levels of certain factors required for infection, thus, rendering them more virulent [83,89]. Third, IAV infection of the lung, through inflammation and oxidative stress, damages the pulmonary epithelium, facilitating pulmonary bacterial colonization and rendering the lung more permissive for subsequent replication. In addition, the adaptive immune response to IAV, mediated by type II and I IFNs produced by anti-viral T cells, impairs both the recruitment of innate immune cells and their ability to kill bacteria [118,119]. The combined tissue damage and compromised immune functions promote systemic spread of *S. pneumoniae* [118,120,121]. As IAV infection alters both the host immune response and bacterial virulence, updated vaccine formulations that maintain protection during co-infections are required.

**Figure 1:**
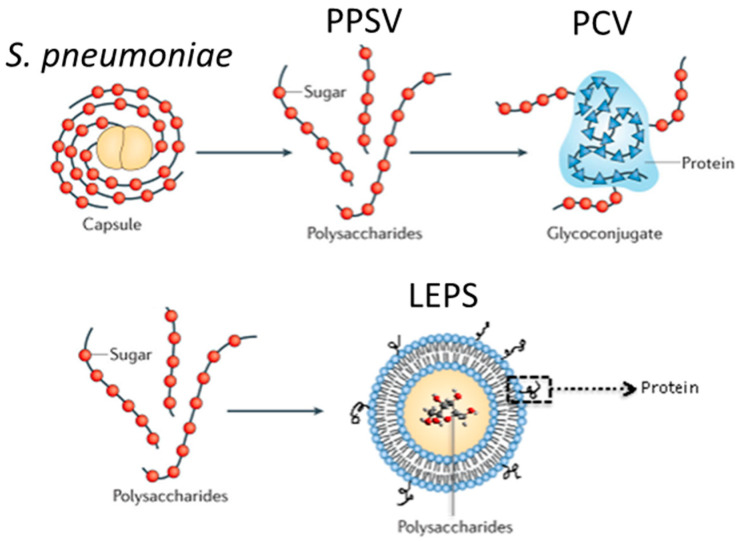
Vaccines for pneumococcal disease derived from polysaccharide content of *S. pneumoniae* to generate either pneumococcal polysaccharide vaccine (PPSV) or pneumococcal conjugate vaccine (PCV) clinical options. The liposomal encapsulation of polysaccharide (LEPS) vaccine platform is presented in comparison.

Built specifically to address weaknesses in PPSV and PCV vaccine options, the liposomal encapsulation of polysaccharide (LEPS) vaccine platform (Figure 1) broadly protects against multiple stages of pneumococcal infection. The LEPS formulation features a liposomal vaccine carrier that encapsulates serotype-specific polysaccharides with the capability to scale to any desired number required for vaccine coverage, which is a technical and economic impossibility with current glycoconjugate formulations. The LEPS vehicle also includes a non-covalent attachment mechanism (via either metal-based chelation or biotin affinity) to affix surface proteins, including CRM197 or new protein antigens that have been identified within virulence progression steps for *S. pneumoniae*, such as proteins temporally displayed by invasive biofilm-dispersed bacteria following influenza co-infection [88,122]. Importantly, this binding mechanism mimics the immunological outcome of Prevnar-13 (i.e., IgM to IgG class switching); triggers Th2, Th1, and Th17 responses (which as indicated above are crucial for overall vaccine effectiveness); and extends recognition to >70 *S. pneumoniae* serotypes due to multiple antigen types (polysaccharide and protein) targeting multiple phases of pneumococcal disease progression (including those traditionally triggered by influenza co-infection) [88,122]. Each feature thus positions the LEPS vaccine as a new and improved option for pneumococcal disease.

## 5. Intranasal Vaccine Delivery for Pneumococcal Disease

One approach that could boost vaccine-mediated immunity against pulmonary infections is to elicit mucosal immune responses, which entails reactivity at the interface of the external environment and the mucus membranes of the respiratory system. This, of course, is a primary motivator for IN vaccine administration. In previous efforts with the LEPS vaccine platform applied towards pneumococcal disease, administration routes had utilized more common intramuscular and subcutaneous injections (with inclusion of the alum adjuvant) [88,122]. Once localized in these locations, the LEPS particles are likely recognized by probing phagocytes, engulfed, and processed for antigen presentation. The LEPS particles may also act in an adjuvant-like manner, activating immune cells, enhancing antigen uptake, and eliciting a more robust immune response.

However, other efforts in pneumococcal disease vaccine research have begun testing the potential for IN administration. In mice, intranasal immunizations with killed [123] or live pneumococci were shown to protect against invasive pneumococcal disease in young hosts [96,124,125,126]. This protection was mediated by both induction of systemic antibody responses [96] as well as mucosal cell-mediated responses including IL-17-producing lung-resident CD4^+^ T cells [124,125,126]. Importantly, in humans, experimental pneumococcal carriage, where live pneumococci are administered intranasally to volunteers, similarly elicited systemic antibody responses [127,128], elicited lung IL-17^+^ CD4^+^ memory T cells [129], stimulated tissue resident innate immune cells [130], and protected against re-colonization by the same serotype [131]. Further, intranasal delivery of pneumococcal protein-based vaccines along with adjuvants protected against invasive disease in mouse models [132]. Thus, intranasal immunizations that trigger systemic and mucosal immune responses are likely viable strategies to elicit host protection against lung infections. Intranasal immunization offers other advantages over traditional immunization administration methods, namely, the lack of injection-driven complications including infections at the administration site and a simple, non-invasive administration that could potentially be self-administered or not require expertise of registered nurses (a plus in remote regions in countries where accessibility is an issue).

There are currently only two licensed intranasal vaccines against influenza A and B viruses, FluMist/Fluenz^®^ (MedImmune, Gaithersburg, MD, USA) and Nasovac^®^ (Serum Institute of India Ltd. Hadapsar, India). Both vaccines consist of live-attenuated strains. Intranasal vaccines against a few other pathogens including SARS-CoV-2, Respiratory Syncytial Virus (RSV) and *B. pertussis* have also reached clinical trials (ClinicalTrials.gov). However, the lack of safe mucosal adjuvants has been an obstacle to successful widespread intranasal vaccinations in humans [133,134]. No mucosal adjuvants have been approved for human use [135], and alum-based adjuvants, commonly used in more traditional vaccine administrations, have shown the potential for several deleterious effects (local tissue irritation, biased immune response) when administered intranasally [136,137]. However, intranasal delivery in the absence of adjuvants may not elicit protective immune responses and could alternatively induce tolerance [138].

Liposomes have shown potential as adjuvants, as have other formulations and additions (such as the inclusion of CpG oligodeoxynucleotide) [137,139,140,141,142,143,144]. As such, vaccine designs that leverage liposomal antigen delivery (such as the LEPS platform introduced above, for example) may very well support efforts in intranasal administration. In doing so, however, liposomal formulations must account for natural forms of bodily defense against intranasal entry of foreign particles, including mucociliary clearance and various barriers to cellular entry [145]. Many liposomal formulation may be prone to these challenges due to a negative surface charge that provides an electrostatic barrier to the interaction with negatively charged mucus and the antigen presenting cells located in the nasal cavity [146]; liposomal vaccine carriers may also lack in mechanical stability when delivered to the nasal passage.

Table 4 summarizes IN administration efforts in more detail for pneumococcal disease application. Here, these studies, all conducted over the last 10 years, show positive IN vaccine efforts for pneumococcal disease. Nearly all of those listed use a direct addition of the antigen content to the nasal region, with only a couple of entries using some sort of material-based formulation to assist in nasal localization and/or immune reactivity. The majority of cases rely upon subunit protein antigens, particularly those that have been identified as a promising marker of virulence. If included, adjuvant content spans non-biologic (liposomal, polymeric, alum) and biologic (chitosan, various bacterial toxins, macromolecules) materials. Though it should be noted that there have been previous concerns of host toxicity associated with bacterial-derived toxoid protein formulations when administered intranasally [133,134].

## 6. Conclusions

Respiratory tract diseases have a long history of affecting human health, with ongoing and recent events emphasizing this historical impact. The pathogens responsible for these diseases span bacterial and viral agents, and though antibiotics have been and continue to be used for the bacterial sources of disease, vaccines have emerged as dominant options for all the main diseases highlighted in this mini-review, spanning influenza, pneumococcal disease, pertussis, tuberculosis, and of course COVID-19. Given the localized disease impact to the pulmonary system, intranasal (IN) vaccine delivery offers a logical option to enhance the eventual immune response to the responsible infectious agents. Here, we have outlined IN utility, prevalence, and approaches for respiratory tract diseases, with an emphasis on vaccine administration for pneumococcal disease, which has a broad impact globally, especially amongst the elderly, and can be particularly synergistic with influenza co-infection. The advantages of IN vaccine delivery may offer new and better vaccine regimens for pneumococcal disease, and several more recent efforts towards this end highlight ongoing approaches that utilize a range of sub-unit and cellular antigenic cargo.

## Figures and Tables

**Table 1:** Main Respiratory Illnesses and Their Treatment Methods.

Disease	Infectious Agent	Notes	Therapeutic/Preventative Options	References
Influenza	Primarily Influenza A virus	Millions of global cases and hundreds of thousands of deaths annually Responsible for historic (Spanish, Asian, Russian, Hong Kong Flu) and more recent (swine and avian flu) outbreaks	Neuraminidase inhibitors (Tamiflu) Seasonal and dedicated vaccines Cap-dependent endonuclease inhibitor	[17]
Pneumococcal Disease	*Streptococcus* *pneumoniae* (bacteria)	Millions of global deaths annually Affects young, elderly, resource-limited groups Commonly synergistic with influenza	Antibiotics Polysaccharide conjugate and non-conjugate vaccines	[18]
Whooping Cough (Pertussis)	*Bordetella* *pertussis* (bacteria)	Part of common DTaP vaccine (diphtheria-tetanus-pertussis) Millions of global cases annually	Antibiotics Vaccine	[19]
Tuberculosis	*Mycobacterium* *tuberculosis* (bacteria)	Global incidence in millions Complicated by latent and drug resistant forms	Antibiotics BCG vaccine	[20]
Coronavirus-based diseases	Various viruses (including SARS-CoV-2)	Previous SARS (2002) and MERS (2012) outbreaks Current COVID-19 pandemic	Recent COVID-19 vaccines Antiviral drugs and advanced therapeutics (including antibody treatments)	[21,22]

**Table 2:** Intranasal Vaccine Delivery for Respiratory Tract Diseases.

Disease	Number of IN Applications (Identified Using the PubMed Search Engine for Indicated Year)				
	2009	2014	2019	2020	2021
Influenza	14	23	17	6	2
Pneumococcal Disease	4	2	9	3	2
Whooping Cough	1	3	0	3	1
Tuberculosis	4	6	3	3	3
COVID-19	N/A	N/A	N/A	6	6

**Table 3:** Range of IN Approaches.

IN Method Used	Rational	Times Tested	Disease Application	Reference
**Dry Powder** -Inulin nanoparticles -Chitosan nanospheres	Highly stable Extended residence time	2	Influenza (Influenza A virus)	[43,44]
**Intranasal Instillation** -Stabilized protein subunit -Nanoparticles -Inert bacterial spores -Bacterium-like particles	Simple and easy form of delivery Low cost	7	Pneumonia (*Actinobacillus pleuropneumoniae*)	[45]
			Tuberculosis (*M. tuberculosis*)	[46,47,48]
			Pneumococcal Disease (*S. pneumoniae*)	[49]
			Influenza (Influenza A virus)	[50,51]
**Aerosolization** -Adenovirus vector-based	Efficient delivery of materials Targets lower respiratory tract	1	Influenza (Influenza A virus)	[52]
**Nasal Gel** -Cationic cholesteryl pullulan	Reduction in nasal clearance Sustained release	1	Pneumococcal Disease (*S. pneumoniae*)	[53]

**Table 4:** Intranasal Vaccine Studies for Pneumococcal Disease.

Vaccine Category	Antigen Content (IN Method)	Adjuvant Included	Reference
Subunit Vaccine	*S. pneumoniae* serotype 4 capsule (Intranasal Instillation)	No adjuvant	[147]
	Recombinant PspA (Nasal Gel)	No adjuvant	[53]
	Recombinant PspA (Intranasal Instillation)	DOTAP/DC-chol liposome	[148]
	Recombinant PspA (Intranasal Instillation)	Chitosan	[149]
	Recombinant PspA (Intranasal Instillation)	*Clostridium perfringens* enterotoxin (C-CPE)	[150]
	Recombinant PspA/BLP (Intranasal Instillation)	No adjuvant	[49]
	Recombinant PspA (nanoparticle absorbed) (Intranasal Instillation)	(PGA-co-PDL) nanoparticles	[151]
	Recombinant PLY (Intranasal Instillation)	Aluminum hydroxide	[152]
	Recombinant LytA (Intranasal Instillation)	CpG oligodeoxynucleotides	[153]
	Recombinant PspA/FlaB fusion (Intranasal Instillation)	Recombinant FlaB	[154]
Live and/or Attenuated	*S. pneumoniae* Y1 (Intranasal Instillation)	Cholera Toxin	[155]
	*S. pneumoniae* serotype 19F/23A/35B (Intranasal Instillation)	No adjuvant	[124]
	*S. pneumoniae* serotype 19F (Intranasal Instillation)	No adjuvant	[125]
	*S. pneumoniae* serotype 23F (Intranasal Instillation)	No adjuvant	[129]
	*S. pneumoniae* serotype 6B (Intranasal instillation)	No adjuvant	[130]
	*S. pneumoniae* serotype 6B (Intranasal instillation)	No adjuvant	[131]
Inactivated/Killed	Gamma irradiated nonencapsulated *S. pneumoniae* TIGR4 (Intranasal Instillation)	Cholera toxin	[156]
	Gamma irradiated whole cell *S. pneumoniae* (Intranasal Instillation)	No adjuvant	[157]

## Data Availability

Not applicable.
